# Prospects for management of whitefly using plant semiochemicals, compared with related pests

**DOI:** 10.1002/ps.5058

**Published:** 2018-06-13

**Authors:** Stefanie Schlaeger, John A Pickett, Michael A Birkett

**Affiliations:** ^1^ Biointeractions and Crop Protection Department Rothamsted Research Harpenden UK; ^2^ School of Chemistry University of Cardiff Cardiff UK

**Keywords:** whitefly, semiochemicals, volatile organic compounds, repellents, olfaction, pest control

## Abstract

Whitefly (Hemiptera: Sternorrhyncha: Aleyrodidae) pests, including the tobacco whitefly, Bemisia tabaci, and the greenhouse whitefly, Trialeurodes vaporariorum, are important economically in agriculture. Whiteflies are controlled mainly by synthetic insecticides but resistance to these is evolving rapidly. A semiochemical‐based management strategy could provide an alternative to the use of insecticides, by exploiting natural volatile signalling processes to manipulate insect behaviour. Whitefly behaviour is affected by differences in plant odour blends. Selected compounds have been suggested as putative semiochemicals, but in only a few studies have potential volatiles been characterized by electrophysiology or olfactometry. Application of antennal preparation methods from closely related families, the aphids (Hemiptera: Aphididae) and psyllids (Hemiptera: Psyllidae), may help to facilitate whitefly electroantennography. Behavioural bioassays are essential to identify the repellent or attractant effect of each semiochemical. The relevance of semiochemicals in whitefly management needs to be evaluated in the respective cultivation system. Although the value of semiochemicals against whiteflies has not been demonstrated in the field, there is an emerging range of possible field applications and some promising prospects. Overall, the olfactory system of whiteflies needs to be elucidated in more detail. © 2018 The Authors. *Pest Management Science* published by John Wiley & Sons Ltd on behalf of Society of Chemical Industry.

## 1 INTRODUCTION

Whiteflies (Hemiptera: Sternorrhyncha: Aleyrodidae), including the tobacco whitefly, *Bemisia tabaci,* and the greenhouse whitefly, *Trialeurodes vaporariorum*, are common agricultural pests that damage a wide range of economically important crop plants, such as tomato (*Solanum lycopersicum*), cucumber (*Cucumis sativus*) and watermelon (*Citrullus lanatus*), particularly by acting as vectors of devastating plant viruses.^1^ Management of whitefly populations relies predominantly on the deployment of broad‐spectrum synthetic insecticides, but because of rapid evolution in insecticide resistance,^2^ new interventions are urgently needed. Semiochemical‐based approaches are considered environmentally benign alternatives to the use of insecticides because semiochemicals act only as signals and are not toxic at the levels deployed. Semiochemicals also have an essential evolutionary role and, although their use in pest management could cause selection for resistance, other related semiochemicals would need to evolve in order to fulfil the crucial signalling role originally targeted. Newly evolved chemicals could be readily identified and used rationally to replace semiochemicals to which resistance had previously evolved.^3^ Semiochemicals are detected by insect olfactory organs, which are mostly located on the antennae. Olfaction was not thought to play a significant role in whitefly host plant selection until the beginning of the 21st century, with research prior to that focusing mainly on whitefly vision. However, increasing numbers of studies have shown that whitefly behaviour is affected by plant‐emitted volatile organic compounds (VOCs). In behavioural bioassays, whitefly preference varies between potential host plants and even among host plant varieties,^4^, ^5^, ^6^ cultivars^4^ and accessions.^7^ Furthermore, whiteflies can discriminate between different qualitative conditions in host plants, for example, differences in nitrogen fertilization,^8^ leaf position,^9^ aphid colonization and virus infection.^10^, ^11^, ^12^ Moreover, the ultrastructure of certain antennal sensilla of *B. tabaci*, *T. vaporariorum* and *Aleyrodes proletella* indicates an olfactory function.^13^, ^14^, ^15^ Odorant‐binding and chemosensory proteins have been detected in *B. tabaci* using transcript analysis,^16^ and certain volatile compounds have been shown to bind to chemosensory proteins of *B. tabaci*.^17^ The whitefly olfactory system is so highly developed that it can even differentiate between stereoisomers of VOCs.^18^

In this review, we focus on plant‐produced, volatile semiochemicals and their potential application in whitefly management. The aim of this review is to identify gaps in whitefly olfaction research and encourage work on this topic. The literature focuses exclusively on economically important whitefly species and biotypes of *B. tabaci* and *T. vaporariorum,* but here they are compared with other hemipterous pests in the suborder of the Sternorrhyncha, the aphids (Hemiptera: Aphididae) and psyllids (Hemiptera: Psyllidae). All adults of these families are phloem‐feeders on host plants and are vectors of plant pathogens.

## 2 IDENTIFICATION AND EVALUATION OF PLANT‐EMITTED WHITEFLY SEMIOCHEMICALS

### 2.1 Isolation of putative semiochemicals

*Bemisia tabaci* and *T. vaporariorum* are extreme generalists and thus must be able to detect the different volatiles specific to many host plants.^19^, ^20^ An expedient approach to identifying whitefly semiochemicals is to compare the volatile collections from different physiological conditions of the host plant that evoke different behavioural responses from whiteflies. For example, VOCs released from less‐attractive plants might be repellent compounds. Furthermore, it is important to include compounds that differ in their proportions because the ratio of compounds can be crucial.^21^, ^22^ Principal component analysis of the volatile collections may be helpful to identify putative semiochemicals within a mixture.^4^, ^23^ Various techniques for collecting plant volatiles are available, such as solvent extraction, steam distillation or air entrainment.^5^ The latter is preferred because headspace collections represent actual released quantities of naturally occurring compounds. In addition, this non‐destructive sampling method does not risk extracting compounds formed by damaged plant tissue. For example, green leaf volatiles are detected only in headspace collections from tomato plants after mechanical wounding.^7^ The technical set‐up of the sampling method, e.g. the choice of adsorbent material, needs to be investigated to achieve the best possible outcome.^24^

### 2.2 Whitefly electrophysiology towards semiochemicals

**Figure 1:**
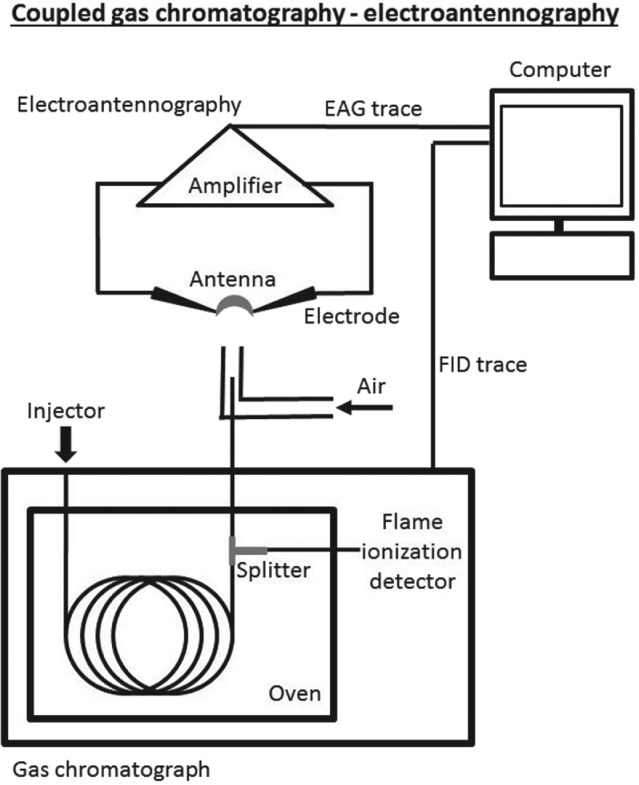
Overview of the technical set up of a gas chromatography–flame ionization detection coupled with electroantennography.

Usually an extract of collected plant volatiles includes a complex and diverse range of compounds, but only a subset of them is likely to have a semiochemical role.^22^ High‐resolution gas chromatography (GC) coupled with electroantennography (GC–EAG) and a detector (e.g. flame ionization detector) is a powerful tool for the identification of semiochemicals within a blend of compounds (Fig. 1). Here, the ability of a compound to be perceived at the olfactory level is indicated by a measured voltage deflection caused by olfactory receptor neurones localized in sensilla on the antennae.^25^ The bioactive compounds are then further identified by GC‐coupled mass spectrometry and/or nuclear magnetic resonance spectroscopy after purification by preparative GC. However, no GC–EAG study with whiteflies has been published to date. Apparently, EAG is not a favoured technique for whiteflies. Among the studies on whitefly olfaction, only two include EAG measurements.^7^, ^18^ In these, excised antenna of *B. tabaci* adults were mounted on a custom‐made holder and volatile terpenoids emitted from less‐preferred accessions of the wild tomato plants *Solanum pennellii, S. habrochaites* and *S. peruvianum* were puffed individually over the antenna. Excised whitefly antennae did not remain viable for the duration of a GC–EAG experiment using a collected plant volatile extract.^18^ Thus, the method is not yet adapted sufficiently for whiteflies to identify active compounds by GC–EAG. Whitefly electrophysiology might learn from the longer ongoing research on aphid olfaction, in which GC–EAG and EAG are used widely to detect semiochemicals.^26^ The longevity of the EAG preparation might be increased by a different antennal preparation technique, for example a whole‐insect preparation. An EAG study on the black bean aphid *Aphis fabae* lasted longer with a whole‐insect preparation than with excised antennae.^27^ Here, the aphid is immobilized using a copper wire restraint. A different and successful approach that involved fixing insects within pipette tips was used for psyllids, where whole‐insect preparations were used for GC–EAG and EAG.^28^ The whole‐insect preparation reduces the risk of drying antennae with the resistance increasing to a level too high for measurements. Another advantage of the longer usable life of the antennal preparation is the option for an extended recovery time between single stimulations. The antennal responsiveness of *A. fabae* was higher with a longer recovery time when using the whole‐insect preparation technique.^27^

A further technique for investigating the olfactory response of insects towards semiochemicals utilizes single sensillum recordings. Here, the electrical activity of one sensillum is measured rather than all sensilla on the antennal flagellum.^29^ This method, alone and in combination with GC, has been used for decades in aphid olfaction research and more recently has been applied successfully in psyllid olfaction research.^23^ This technique can help to receive antennal responses when there is a low number of sensilla, as shown in the carrot psyllid Trioza apicalis.^30^ No absolute number of antennal sensilla was presented in B. tabaci biotypes, but the study indicates a similar sparse sensillar set‐up compared with T. apicalis.^13^, ^30^, ^31^

Overall, research on whitefly olfaction would benefit from more electrophysiological studies. GC–EAG analysis of a volatile collection can greatly facilitate the search for semiochemicals. EAG‐active VOCs are detected by insect antennae and most likely affect whitefly behaviour. The EAG method can also be used for dose–response tests comparing antennal responses at different doses of the same semiochemical. This investigation can help to identify the most efficient dose for whitefly management.

### 2.3 Evaluation of whitefly behaviour towards semiochemicals

**Figure 2:**
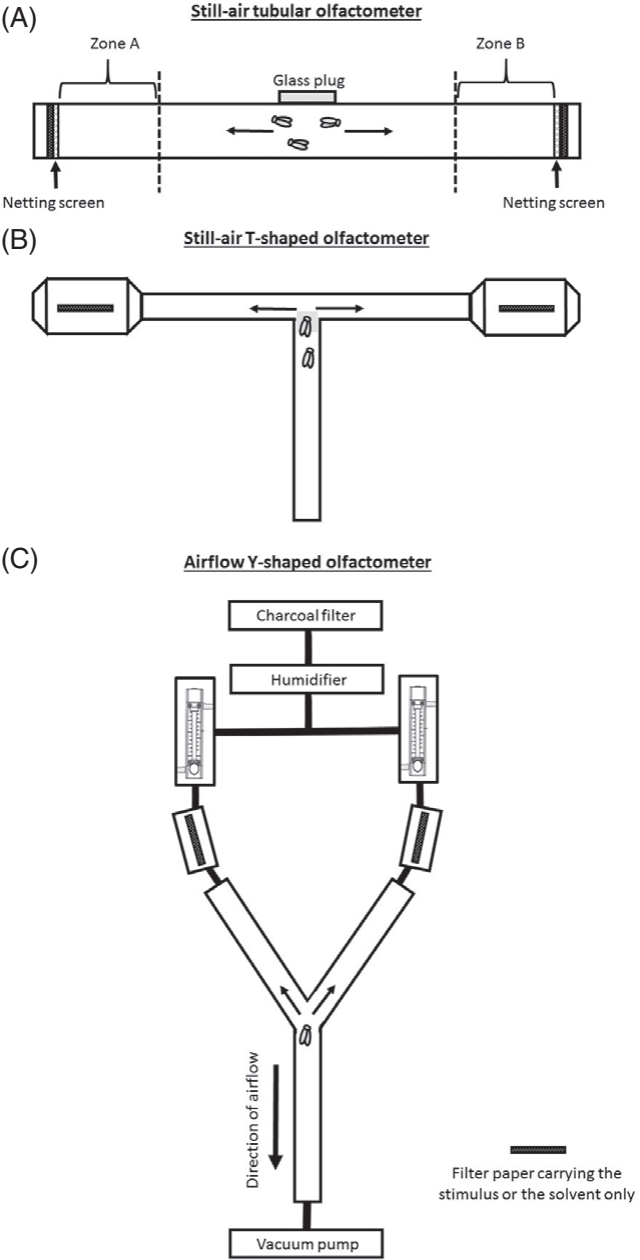
Designs of different dual‐choice olfactometers. The directions for the whitefly's choices and the locations of the stimuli/control are illustrated.

EAG studies do not reveal the behavioural activity of an identified plant‐emitted semiochemical, i.e. whether it is a repellent or an attractant. For this, insect behaviour towards olfactory cues needs to be evaluated, for example in an olfactometer assay. Olfactometers are sealed devices with a system of channels with or without directed airstreams providing different odour sources. The insect is released into the system where it can move freely and decide between channels permeated with the test odour and with the solvent or with air alone. A solvent is often needed to apply semiochemicals to the release device (usually filter paper) and serves as a control. Evaluation of insect behaviour can be based on the choice of channel or on the time spent in a particular channel. It is expected that the insect will respond positively to channels containing an attractant stimulus compared with solvent/air, and positively to channels containing solvent/air compared with a repellent stimulus. There is wide variation in the procedure for measuring whitefly responses to semiochemicals (e.g. the number of whiteflies released, the dimensions of the olfactometer or the evaluation method used), making it difficult to compare studies. However, dual‐choice olfactometers (Fig. 2), in which the insect chooses between two channels (one containing the stimulus and one containing only the solvent or air), have been used in all tests with whiteflies responding to individual VOCs (Table 1). A tubular olfactometer is a linear device in which whiteflies are released into the middle of the tube and can move freely in either direction (Fig. 2A). The behavioural bioassay is conducted without airflow. The tested airborne semiochemicals have been considered as repellents using the avoidance index as the evaluation method (Table 1). This index is the number of whiteflies in the repellent zone subtracted from the number of whiteflies in the control zone divided by the total number of whiteflies counted. Bioassays in the T‐shaped olfactometer are conducted without an airstream (Fig. 2B). Whiteflies are released at the base of the device and need to move via the junction into one of the branches. Their choice is evaluated by the number of individuals in each of the decision chambers. The Y‐shaped olfactometer bioassay is performed using a directed, charcoal‐filtered and humidified airstream (Fig. 2C). Here, whiteflies are also released at the base of the olfactometer. They need to walk to the junction and make a choice. Their preference is evaluated by counting the number of individuals that entered the respective channel in a defined way (e.g. moving at least one third into the respective channel). Repellency olfactometer tests predominate because of the greater interest in preventing the settlement of viruliferous whiteflies on crop plants (Table 1). Two types of olfactory‐mediated repellents have been defined depending on their effect on insect behaviour.^38^ So‐called true repellents cause insects to actively move away from the odour source, whereas odour‐masking repellents reduce or disrupt the attractiveness of the host plant. Investigation of true repellents using a Y‐shaped olfactometer might be difficult because whiteflies may not enter the choice region.^38^ This might be resolved by including the response rate for all tested whiteflies (responders vs. non‐responders) or excluding responses below a set minimum from the evaluation.^36^, ^37^ However, none of the studies has addressed whitefly behaviour towards a true repellent by evaluating an oriented movement away from the odour source. Overall, whitefly repellents or attractants are evaluated based on the proportion of responding whiteflies in relation to the solvent used or clean air. The degree of the semiochemical effect on whitefly behaviour is also dose‐dependent.^5^, ^6^ The terms repellency and attractancy should be used with caution and always in context.

**Table 1:** Overview of olfactometer tests evaluating the behavioural response of Bemisia tabaci towards plant‐produced, individual volatile organic compounds

Type of olfactometer	Compound	Stated effect	Evaluation method	Reference
T‐shaped	(*R*)‐Limonene	Attractancy	Preference	5
T‐shaped	(*E*)‐Caryophyllene	Attractancy	Preference	5
Tubular	(*R*)‐Limonene	Repellency	Avoidance index	6
Tubular	Myrcene	Repellency	Avoidance index	6
Tubular	(*E*)‐Ocimene	Repellency	Avoidance index	6
Tubular	(*R*)‐Limonene	Repellency	Avoidance index	32
Tubular	Limonene*	Repellency	Avoidance index	32
Tubular	Citronellal	Repellency	Avoidance index	32
Tubular	Citral	Repellency	Avoidance index	32
Tubular	α‐Pinene*	Repellency	Avoidance index	32
Tubular	Geranyl nitrile	Repellency	Avoidance index	32
Y‐tube	2‐Ethyl‐1‐hexanol*	Attractancy	Preference	33
Y‐tube	o‐Xylene	Repellency	Preference	33
Y‐tube	Phenol	Attractancy	Preference	33
Y‐tube	α‐Pinene*	Repellency	Preference	33
Y‐tube	Salicylic acid	Repellency	Preference	34
Y‐tube	Limonene*	Repellency	Preference	34
Y‐tube	1,8‐Cineole	Repellency	Residence time	35
Y‐tube	Linalool*	Attractancy	Residence time	35
Y‐tube	(*E*)‐2‐Hexenal	Attractancy	Response/attraction rate	36
Y‐tube	3‐Hexen‐1‐ol*	Attractancy	Response/attraction rate	36
Y‐tube	Limonene*	Repellency	Response/attraction rate	36
Y‐tube	(*R*)‐Limonene	Repellency	Response	37
Y‐tube	Geranyl nitrile	Repellency	Response	37

* Isomeric composition not given.

**Figure 3:**
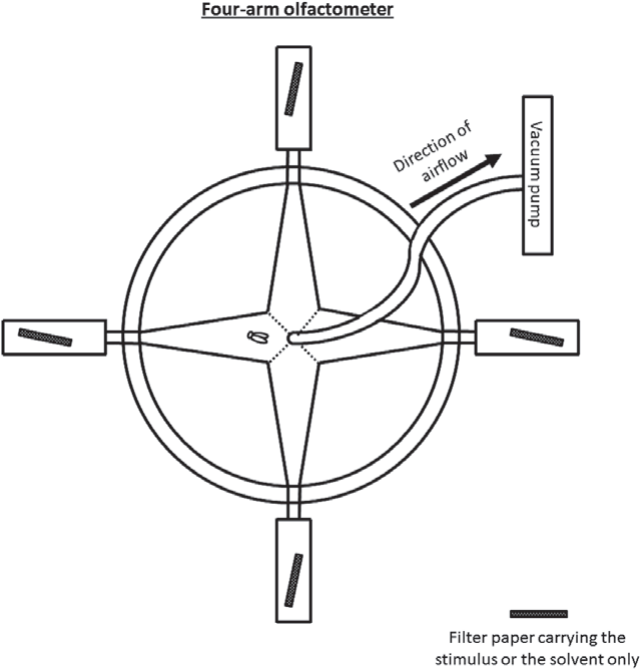
Design of a four‐arm olfactometer. The four areas of the arena for the whitefly's choices and the locations of the stimuli/control are illustrated.

The four‐arm olfactometer (Fig. 3) is a different device in insect olfactometry and a standard bioassay in aphid studies used for either repellents and attractants.^39^, ^40^ In this experimental set‐up, the insect can move freely within an arena divided into four areas with respective airstreams. There have been 2 four‐arm olfactometer tests with *B. tabaci* published to date. These investigate the effect of odour masking of non‐host plants or aphid‐induced plant volatiles rather than selected semiochemicals.^10^, ^41^ The four‐arm olfactometer might have received little consideration for whitefly bioassays because its design requires insects to actively explore all arms. Whiteflies are known to fly over even short distances. For example, they fly up the host plant when disturbed. However, they may actively walk in a four‐arm or Y‐shaped olfactometer (Schlaeger S, personal observation).

In contrast to evaluation of a true repellent, experimental set‐ups for host odour‐masking repellents should include volatiles emitted by the host plant. In addition, the crop plant has visual cues that might also interfere with the effect of the semiochemical on whitefly behaviour. For instance, the visual attractiveness of the crop plant might override the effect of a host odour‐masking repellent. In general, all potential semiochemicals must be tested in the respective cultivation system for their relevance in whitefly control. Under field conditions, whitefly semiochemicals are exposed to all types of odorants from the environment. The effect of a whitefly semiochemical might be diminished in a mixture of VOCs.

An indirect but important approach to control whiteflies is to identify plant‐mediated semiochemicals that attract natural enemies. For example, (*Z*)‐3‐hexen‐1ol, (*E*)‐4,8‐dimethyl‐1,3,7‐nonatriene (DMNT) and 3‐octanone are emitted in higher quantities from *T. vaporariorum*‐infested bean plants, *Phaseolus vulgaris*, than from plants that are not infested.^42^ Synthetic versions of these VOCs, when presented individually or in a blend, increased the attractancy of the whitefly parasitoid, *Encarsia formosa,* in wind tunnel bioassays. *Arabidopsis thaliana* plants emit myrcene when attacked by *B. tabaci*.^43^ In a Y‐shaped olfactometer, application of synthetic myrcene to the odour of non‐infested *A. thaliana* plants attracted more *E. formosa* parasitoids than the odour of plants that were not treated with the semiochemical.^43^

## 3 POSSIBLE APPLICATION OF SEMIOCHEMICALS TO WHITEFLY MANAGEMENT

Keeping whitefly infestation on crop plants below an economic threshold level is part of an integrated pest management strategy. The settlement and feeding of one individual on a crop plant is enough for virus transmission because the vectored virus diseases are systemic, affecting the whole plant. Thus, the economic threshold is very low. Consequently, the odour‐masking effect of a repellent is not sufficient, because whiteflies may still land on the crop plant. To control whitefly virus vectors, a true repellent that prevents whitefly colonization almost completely is preferred.

Semiochemicals are an important component of push–pull technology that combines repellents and attractants within the same cropping system. The pest is deterred from the crop plant (push) and at the same time lured to a more attractive source (pull).^44^ This strategy is especially suited to the control of greenhouse pests, such as *T. vaporariorum*, because of the confined space involved.^44^

Semiochemicals can be integrated into a cropping system using cultural practices, e.g. intercropping with semiochemical‐emitting plants. Adult *B. tabaci* infestation of tomato plants was reduced by intercropping with either coriander (*Coriandrum sativum*) or Greek basil (*Ocimum minimum*) plants, or a citronella grass (*Cymbopogon* spec.) mulch.^45^ Where economically viable, another possibility for field application of active semiochemicals is the deployment of synthesized VOCs *via* sprays or slow‐release dispensers.^44^ No field studies with selected whitefly semiochemicals have been reported. However, *B. tabaci* settlement on tomato plants was reduced in a greenhouse experiment using bottles with a 1% mixture of (*R*)‐limonene, citral and, as a slow release agent and antioxidant, olive oil (in a ratio of 63 : 7 : 30) as the ‘push’ treatment, and yellow sticky traps as the pull treatment.^32^ Application of the sesquiterpene hydrocarbon and aphid alarm pheromone (*E*)‐β‐farnesene or methyl salicylate in a paraffin oil formulation released from a rubber septum in a wheat (*Triticum aestivum*) field centred on a wheat–pea (*T. aestivum–Pisum sativum*) strip intercropping system reduced aphid infestation and increased the number of parasitized aphids.^46^ The potential of using a push–pull strategy for management of the Asian citrus psyllid *Diaphorina citri*, the vector of *Candidatus Liberibacter asiaticus*, the causative agent of citrus greening disease, has been reviewed.^47^ It has been stated that more knowledge about psyllid–host interactions needs to be generated before more applied studies can be performed. However, potential psyllid semiochemicals have been identified, for example, the homoterpenes DMNT and (*E,E*)‐4,8,12‐trimethyltrideca‐1,3,7,11‐tetraene (TMTT). A synthetic mixture of DMNT and TMTT reduced the attractiveness of the hosts orange jasmine*, Murraya paniculata* and sweet orange Pera D6, *Citrus sinensis*, in a four‐arm olfactometer bioassay.^48^ In Y‐shaped and four‐arm olfactometer assays, dimethyl disulfide, identified from the non‐host guava, *Psidium guajava*, reduced the attractiveness of volatiles from *C. sinensis*.^49^ Furthermore, dimethyl disulfide released from polyethylene vials reduced *D. citri* infestation in an orchard of Valencia oranges, *C. sinensis*, for up to 4 weeks.

A different approach to the deployment of semiochemicals is to modify the emitted VOCs of the crop plant by genetic engineering, such that the plant is either not attractive to pest insects (odour masking) or becomes repellent.^50^ This mode of direct pest management can be supplemented with the attraction of beneficial natural enemies for conservation biological control.^51^ The most prominent example of this strategy to date is the engineering of elite wheat to release (*E*)‐β‐farnesene, which has been confirmed in laboratory bioassays.^52^ However, the repellent effect on aphids could not be confirmed in field studies. The missing effect in the field might be due to bad weather conditions during the trial. Another explanation might be the difference between release of (*E*)‐β‐farnesene from the plant (emitted continuously) and release from the aphid (sudden burst release). Plant glandular trichomes are sources of semiochemicals, making them targets for genetic engineering in pest resistance.^53^ A promising attempt to modify semiochemical biosynthesis in trichomes for whitefly management has been shown in cultivated tomato.^54^ Wild tomato *S. habrochaites* accession PI127826 is naturally less attractive to *B. tabaci* and releases distinct quantities of the sesquiterpene hydrocarbon 7‐epizingiberene.^7^, ^18^ Application of 7‐epizingiberene to cultivated tomato reduced settlement of *B. tabaci* adults in a free‐choice bioassay.^18^ Introduction of the biosynthetic pathway of 7‐epizingiberene into the glandular trichomes of the cultivated tomato with trichome specific promotors led to production of this whitefly semiochemical.^54^ The repellent property against *B. tabaci* was not investigated with the transgenic lines in this study.

Semiochemicals can be expensive to synthesize and may be chemically unstable, which is unfavourable for field application. A possible approach to overcome these challenges is the rational design of analogues of semiochemicals using chemoenzymatic synthesis. In this method, acceptance of the unnatural substrate by the specifically responsible biosynthesis enzyme leads to analogues of the natural product that might have superior properties. Successful use of this synthetic biology approach has recently been demonstrated for the aphid sesquiterpene semiochemical (*S*)‐germacrene D and has led to the rational discovery of novel semiochemicals.^55^ This approach is now being tested with the whitefly semiochemical 7‐epizingiberene and its biosynthesis enzyme, epizingiberene synthase. Biosynthetic pathways for production of the new analogues have the potential of being engineered into crop plants and therefore the library of semiochemical tools for whitefly management widened. The availability of a wide range of tools provides an opportunity to mitigate the evolution of whitefly resistance to semiochemicals.^3^

## 4 CONCLUSION

Plant‐produced VOCs can alter whitefly behaviour, but few studies have investigated the effects of the actual putative semiochemicals through electrophysiology and behavioural work with whiteflies. More information about the olfactory system of whiteflies is needed, for example which sensilla are responsible for olfaction. This knowledge is necessary to identify semiochemicals for subsequent use in whitefly management.

For a better general understanding, it might be useful to broaden the research on whitefly olfaction to other species of economic importance in addition to *B. tabaci*. Few studies deal with *T. vaporariorum* or *A. proletella* although *A. proletella* is of particular interest because it is a specialist in comparison with the generalists *B. tabaci* and *T. vaporariorum*, feeding primarily on cruciferous plant species. In addition, semiochemical interventions against *Trialeurodes* species could be more advantageous because of the higher value of glasshouse products in comparison with arable production.
